# Interferon regulatory factor transcript levels correlate with clinical outcomes in human glioma

**DOI:** 10.18632/aging.202915

**Published:** 2021-04-26

**Authors:** Jin Lei, Ming-Hui Zhou, Fu-Chi Zhang, Kang Wu, Sheng-Wen Liu, Hong-Quan Niu

**Affiliations:** 1 Department of Neurosurgery, Tongji Hospital, Tongji Medical College, Huazhong University of Science and Technology, Wuhan, China

**Keywords:** interferon regulatory factor, expression, prognosis, biomarker, bioinformatic analysis

## Abstract

Members of the interferon regulatory factor (IRF) gene family are crucial regulators of type I interferon signaling, which may play a role in the resistance of glioma to immune checkpoint blockade. However, the expression profiles, potential functions, and clinical significance of IRF family members remain largely unknown. Here, we examined IRF transcript levels and clinicopathological data from glioma patients using several bioinformatic databases, including ONCOMINE, GEPIA, TCGA, and cBioPortal. We found that IRF1, IRF2, IRF5, IRF8 and IRF9 were significantly upregulated in glioma compared to normal brain tissue. Higher IRF1, IRF2, IRF3, IRF4, IRF5, IRF7, IRF8 and IRF9 mRNA levels correlated with more advanced tumor grades and poorer outcomes. Moreover, although IRFs mutation rates were low (ranging from 0.5% to 2.3%) in glioma patients, genetic alterations in IRFs were associated with more favorable patient survival. Functional analysis showed that IRFs participated in glioma pathology mainly through multiple inflammation- and immunity-related pathways. Additionally, correlations were identified between IRFs and infiltration of immune cells within glioma tissues. Collectively, these results indicate that IRF family members, including IRF1, IRF2, IRF5, IRF8 and IRF9, may serve as prognostic biomarkers and indicators of immune status in glioma patients.

## INTRODUCTION

Glioma is the most prevalent primary malignancy in the human brain and is characterized by high recurrence and lethality rates [1]. According to WHO guidelines, glioma is typically divided into low-grade glioma (LGG; grade II and III) and glioblastoma (GBM; grade IV) based on the degree of malignancy [2]. The overall prognosis for glioma patients, especially those with GBM, is poor; median survival is less than two years even after standard treatments, which include surgical resection, chemotherapy, and radiation therapy [3]. More recent studies have investigated the use of novel therapeutic modalities like immunotherapy to treat glioma due to success achieved in several other solid tumors. However, glioma is resistant to monotherapy with immune checkpoint inhibitors (mainly PD-1/PD-L1 blockade), indicating an urgent need to explore the mechanisms of resistance and to identify additional targets for combination therapy [4].

Recent reports suggest that type I interferon signaling, which is regulated by interferon regulatory factors (IRFs), plays an important role in glioma resistance to immune checkpoint blockade [5, 6]. The IRF family consists of nine members, IRF1 to IRF9, all of which possess a well-conserved N-terminal DNA-binding domain (DBD) [7]. They participate in a variety of biological processes including antiviral inflammation, cell proliferation, cell apoptosis, and immune cell maturation, and therefore can participate in both immunity and oncogenesis [8, 9]. IRFs are presumed to play a complex and essential role in glioma pathology and immune microenvironment. Among the nine members, IRF7, the master regulator of transcriptional activation of type I interferon genes, was found to be over-expressed in glioma cells and specimens, promoting microglia recruitment and tumor growth by increasing expression of inflammatory cytokines [10]. In contrast, IRF3, activation of which exerts strong effects on IL-1/IFNγ-induced inflammatory gene expression and suppresses glioma migration and invasion, is downregulated in glioblastoma cells [11]. Furthermore, Liang et al. reported that IRF1 deletion decreased autophagy and increased apoptosis in glioma cell lines, which increased glioblastoma resistance to antiangiogenic therapy [12]. However, the overall expression profile of all nine IRFs, as well as their potential functions in glioma development and distinct clinical significance, has not been fully characterized.

Recent advances in gene sequencing technology have enabled comprehensive analysis of IRF family members with existing bioinformatic tools. In this study, we performed an in-depth exploration of the expression patterns of IRF family members in glioma and evaluated their potential as prognostic biomarkers with the goal of improving molecular diagnosis and prognostic prediction for glioma patients.

## RESULTS

### Gene expression of IRF family members in glioma patients

**Figure 1:**
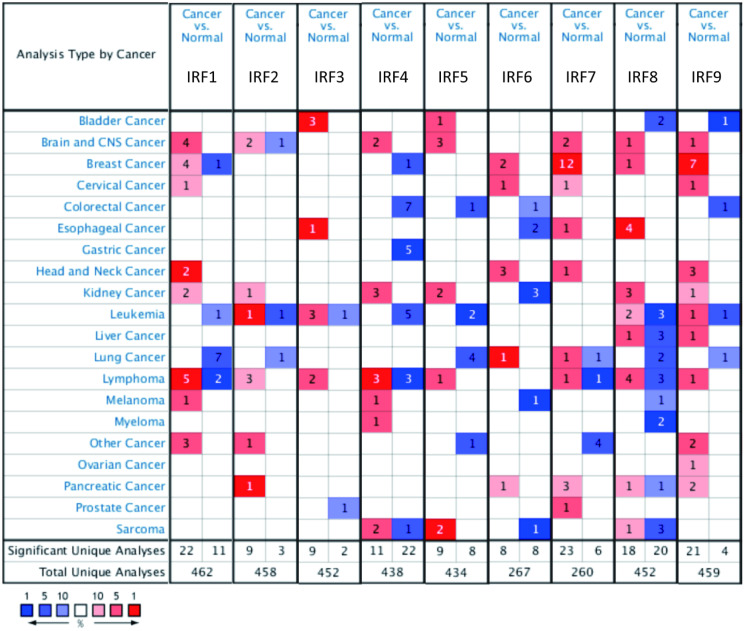
**Transcript levels of the nine IRF family members in different types of cancer (ONCOMINE).** The value inside each box indicates the number of datasets with statistically significant IRF hyper- (red) or hypo-expression (blue). Differences were compared using STUDENT’S *t*-test. *p*-value: 0.05, fold change: 2, gene rank: 10%, data type: mRNA.

IRF family member transcript levels were evaluated in glioma patients using ONCOMINE and GEPIA. As shown in Figure 1, expression of IRF mRNAs was generally upregulated in 20 common human cancers compared to normal tissues according to ONCOMINE data. IRF1, IRF2, IRF4, IRF5, IRF7, IRF8, and IRF9 expression was higher in brain and CNS tumors than in normal brain tissue; no differences were observed for IRF3 and IRF6. In particular, IRF1 expression was 33.893-fold higher in malignant glioma (*p* = 0.008); two additional studies by Liang and Bredel found that IRF1 expression was increased 2.225- and 2.151-fold, respectively, in glioblastoma. IRF2 transcript levels were also higher in glioblastoma than normal brain tissues in two datasets from TCGA (fold change = 3.705 and 2.448, respectively; *p* = 0.002 and 7.92E-9, respectively). The results of Sun’s study suggested that IRF5 was increased 2.125-fold in diffuse astrocytoma (*p* = 1.33E-4) and 2.180-fold in anaplastic astrocytoma (*p* = 3.20E-5). Moreover, studies by Lee, Ramaswamy, and Bredel all found that IRF8 and IRF9 levels were significantly increased in glioblastoma or anaplastic oligoastrocytoma (Table 1).

**Table 1:** Significant changes in IRF mRNA expression among various types of glioma and normal brain tissues (ONCOMINE).

**Gene**	**Type of glioma vs. normal**	**Fold Change**	***t*-test**	***p*-value**	**Study**
IRF1	Malignant Glioma	33.893	3.136	0.008	Pomeroy
	Glioblastoma	2.225	4.455	0.005	Liang
	Glioblastoma	2.151	6.682	3.01E-5	Bredel
IRF2	Glioblastoma	3.705	5.493	0.002	TCGA Brain
	Brain Glioblastoma	2.448	15.072	7.92E-9	TCGA Brain
IRF3	NA				
IRF4	NA				
IRF5	Diffuse Astrocytoma	2.125	5.056	1.33E-4	Sun
	Anaplastic Astrocytoma	2.180	4.539	3.20E-5	Sun
IRF6	NA				
IRF7	NA				
IRF8	Glioblastoma	5.471	11.360	6.64E-5	Lee
IRF9	Glioblastoma	2.420	5.460	3.18E-5	Ramaswamy
	Anaplastic Oligoastrocytoma	2.256	4.830	0.002	Bredel

Abbreviation: NA = not applicable.

**Figure 2:**
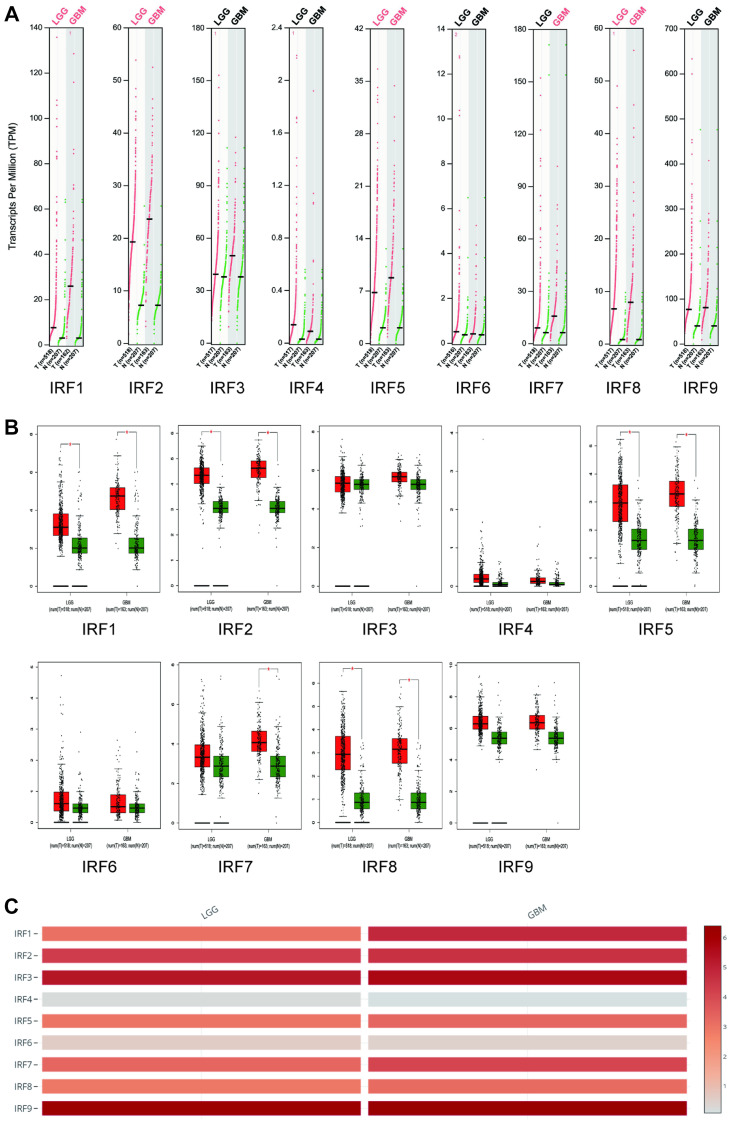
**Transcript levels of IRF family members in LGG and GBM (GEPIA).** The expression profiles (**A**) and box plots (**B**) show that IRF1, IRF2, IRF5, and IRF8 expression were significantly elevated in both LGG and GBM, while IRF7 expression was increased in GBM only. (**C**) IRF4 and IRF6 are the lowest among all IRFs in both LGG and GBM.

We next examined IRF expression in the different glioma subtypes, i.e., LGG and GBM, using GEPIA analysis. IRF1, IRF2, IRF5, and IRF8 expression was significantly higher in both LGG and GBM than in normal tissues, while IRF7 levels were significantly upregulated in GBM tissues only (Figure 2A–2B). Among the nine IRF family genes, IRF4 and IRF6 expression were lowest in LGG and GBM (Figure 2C).

### Correlations between IRF expression and pathological and prognostic parameters of glioma

**Figure 3:**
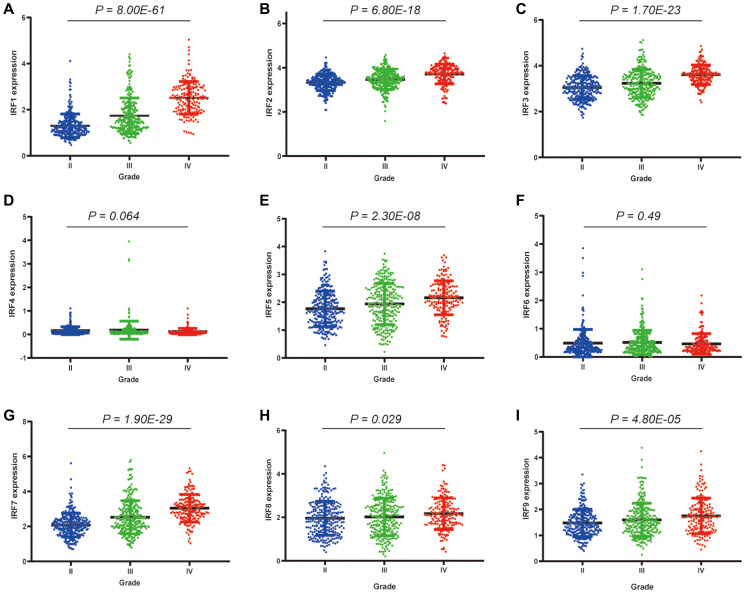
**Correlations between differentially expressed IRF family members and pathological grade in glioma patients.** ^*^P < 0.05, ^**^P < 0.01, ^***^P < 0.001. (**A**) IRF1, (**B**) IRF2, (**C**) IRF3, (**D**) IRF4, (**E**) IRF5, (**F**) IRF6, (**G**) IRF7, (**H**) IRF8, (**I**) IRF9.

Relationships between IRF family member expression and clinicopathological parameters of glioma patients were examined using data from the TCGA database. Among the 260 grade II, 267 grade III, and 173 grade IV glioma patients, significant correlations were observed between IRF1 (*p* = 8.00E-61), IRF2 (*p* = 6.80E-18), IRF3 (*p* = 1.70E-23), IRF5 (*p* = 2.30E-08), IRF7 (*p* = 1.90E-29), IRF8 (*p* = 0.029), IRF9 (*p* = 4.80E-05) expression and pathological grade (Figure 3). There was also a trend towards a correlation between IRF4 expression and pathological grade (*p* = 0.064). No correlation between IRF6 expression and pathological grade was observed (*p* = 0.49). For all of the identified correlations, expression increased as tumors progressed, suggesting that IRFs may play a role in glioma tumorigenesis and progression.

**Figure 4:**
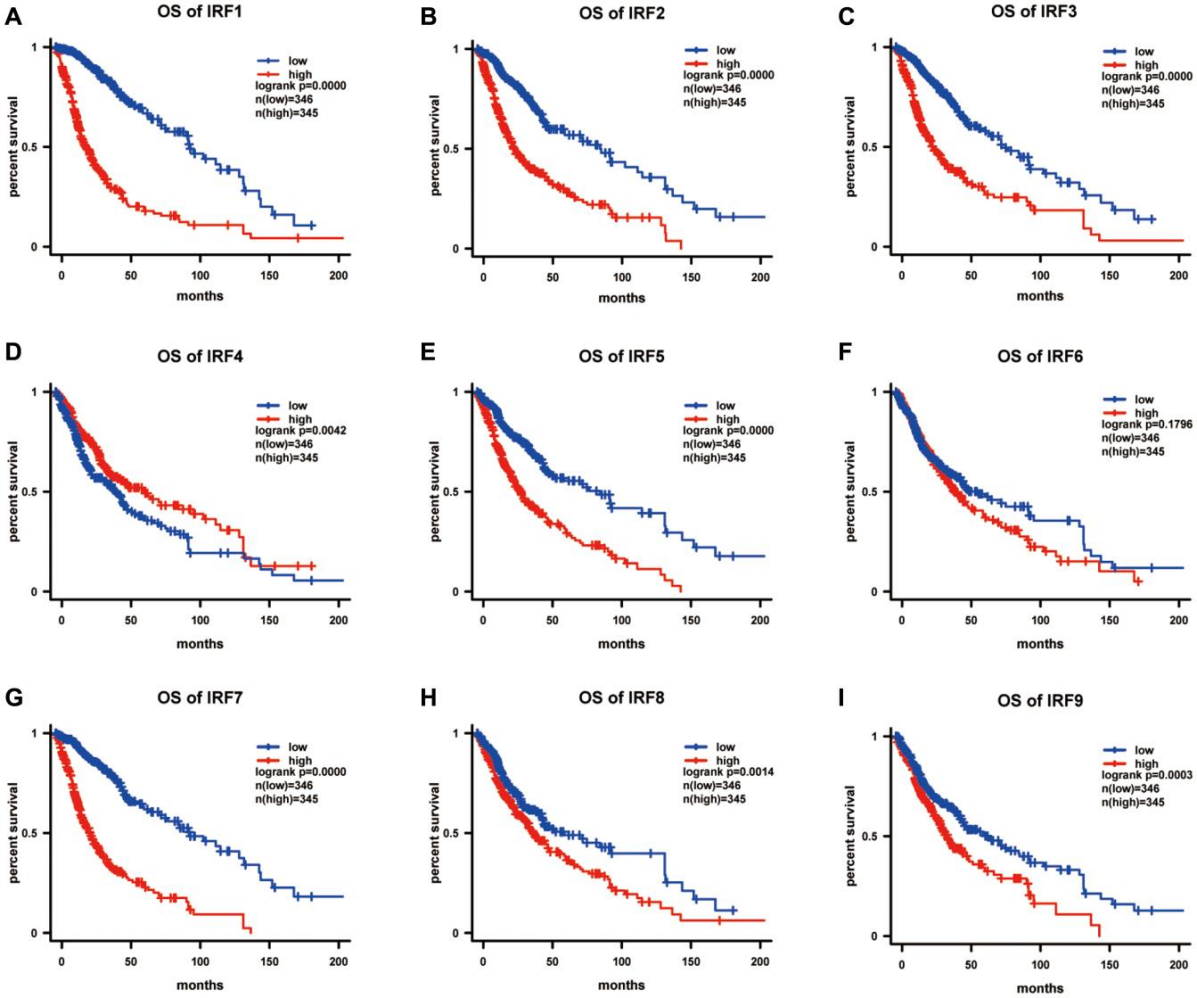
**The prognostic value of IRF family members in predicting overall survival of glioma patients (OS).** The survival curves for (**A**) IRF1, (**B**) IRF2, (**C**) IRF3, (**D**) IRF4, (**E**) IRF5, (**F**) IRF6, (**G**) IRF7, (**H**) IRF8, and (**I**) IRF9 in glioma using the Kaplan-Meier method. ^*^P <0.05.

We further explored associations between IRF expression and survival in glioma patients using Kaplan-Meier analysis. Overall survival curves indicated that glioma patients with lower IRF1 (*p* = 4.42E-33), IRF2 (*p* = 2.22E-16), IRF3 (*p* = 1.43E-15), IRF4 (*p* = 0.004), IRF5 *p* = 5.84E-12), IRF7 (*p* = 6.85E-28), IRF8 (*p* = 0.001), and IRF9 (*p* = 0.000) transcript levels had significantly longer overall survival times (Figure 4).

### Genetic alteration, co-expression, and interaction analysis of IRF family members in glioma

**Figure 5:**
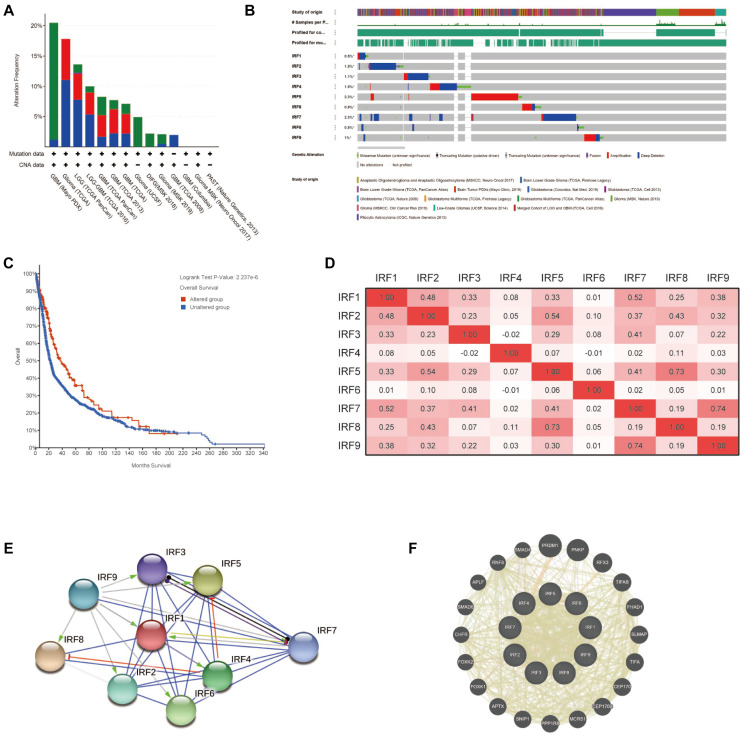
**Genetic alterations, co-expression, and interaction analysis of IRF family members in glioma patients.** (**A**) Summary of genetic alterations in IRF family members in glioma. (**B**) OncoPrint visual summary of alterations in a query of IRF family members (cBioPortal). (**C**) Kaplan-Meier plot comparing overall survival in cases with/without IRF alterations (cBioPortal). (**D**) Correlation heat map of differentially expressed IRF family members in glioma (TCGA glioma dataset). (**E**) Protein-protein interaction network among the nine differentially expressed IRF family members (STRING). (**F**) Top 20 external genes functionally related to IRF family members and the interaction network (GeneMANIA).

To understand the molecular characteristics of IRF family members in glioma, we systematically evaluated genetic alteration, co-expression, and protein interaction networks using multiple tools, including cBioPortal, TCGA, STRING, and GeneMANIA. First, genetic alterations of IRFs in glioma patients were examined using cBioPortal. Among 5504 samples from 5300 patients in 14 glioma datasets, the overall alteration frequency of IRF genes ranged from 1.97% (4/203) to 20.48% (17/83); mutations, deep deletions, and amplification were the most common types of alteration (Figure 5A). For each individual gene, alteration frequencies varied from 0.5% to 2.3% (IRF1, 0.5%; IRF2, 1.5%; IRF3, 1.1%; IRF4, 1.5%; IRF5, 2.3%; IRF6, 0.9%; IRF7, 2.3%, IRF8, 0.5%; IRF9, 1%) (Figure 5B). We further assessed the impact of IRF gene alterations on prognosis and found that glioma patients with alterations exhibited a longer overall survival compared with those without alterations (*p* = 2.237E-6) (Figure 5C).

We next explored potential co-expression among IRF family genes using data from TCGA glioma dataset. Pearson's correlation results revealed significant positive correlations between the following IRFs: IRF1 with IRF2 (r = 0.48) and IRF7 (r = 0.52); IRF2 with IRF1, IRF5 (r = 0.54), and IRF8 (r = 0.43); IRF3 with IRF7 (r = 0.41); IRF5 with IRF7 (r = 0.41) and IRF8 (R = 0.73); IRF7 with IRF9 (r = 0.74) (Figure 5D). Little correlation was observed between IRF4, IRF6, and the rest of genes in the family.

Next, we conducted a network analysis to examine potential internal interactions among IRF family genes as well as external interactions with other functionally related genes. PPI network analysis using STRING software revealed close protein-protein associations among the IRF family genes with 9 nodes, 32 edges, and an average node degree of 7.11 (*p* < 1.0E-16; Figure 5E). Also, GeneMANIA analysis revealed that PRDM1, PNKP, RFX3, TIFAB, FHAD1, SLMAP, TIFA, CEP170, CEP170B, MCRS1, PPP1R8, SNIP1, APTX, FOXK1, FOXK2, CHFR, SMAD6, APLF, RNF8, and SMAD4 were primarily associated with the modulation and function of IRF genes in glioma (Figure 5F). Additionally, GeneMANIA analysis indicated that all IRF family members shared protein domains, and IRF1, IRF2, IRF3, IRF7, IRF8, and IRF9 were colocalized within cells.

### Functional enrichment analysis of IRF family members in glioma

**Figure 6:**
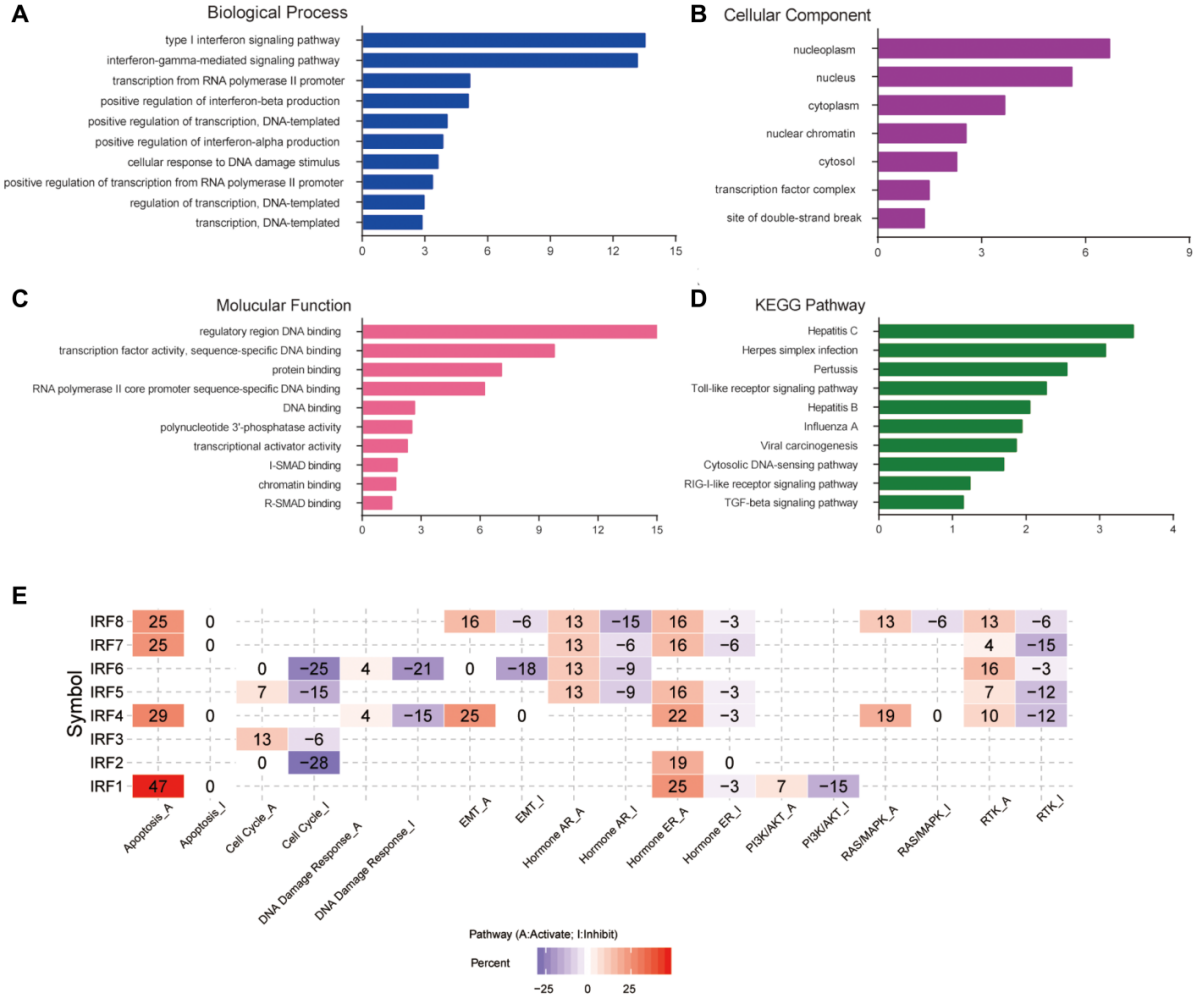
**Functional enrichment and cancer-related pathway analysis of IRF family members in glioma.** Bar plots of GO enrichment terms in (**A**) biological process, (**B**) cellular component, and (**C**) molecular function. (**D**) Bar plot of KEGG enrichment terms. (**E**) Heat map of cancer pathway activity of IRF family members in glioma (GSCALite).

DAVID software was used to analyze the biological functions of differentially expressed IRF family members and their functionally related genes. A total of 55 GO items (BP: 32; CC: 7; MF: 16) and 11 KEGG items were enriched. Figure 6A–6D shows the top 10 most highly enriched items for each category. Among the 10 most highly enriched functions in the BP category, type I interferon and interferon-gamma-mediated signaling pathways, transcription from RNA polymerase II promoter, positive regulation of transcription, DNA-templated, cellular response to DNA damage stimulus, and negative regulation of cell proliferation were associated with glioma tumorigenesis and progression (Figure 6A). In the CC category, IRFs and their functionally related genes were mainly enriched in the nucleoplasm, nucleus, cytoplasm, and transcription factor complex (Figure 6B). The most enriched GO terms in the MF category were regulatory region DNA binding, transcription factor activity, and protein binding (Figure 6C). The main pathways enriched in KEGG analysis were several virus infection pathways, viral carcinogenesis pathways, the toll-like receptor signaling pathway, and the TGF-beta signaling pathway (Figure 6D). We also examined predicted roles of the IRF family in noted cancer-related pathways using GSCALite. The results showed that the IRF family may modulate glioma by activating apoptosis, activating the EMT, hormone ER, RAS/MAPK, and RTK pathways, and by inhibiting cell cycle, DNA damage response, and the PI3K/AKT pathway (Figure 6E).

### Immune infiltrate analysis of IRF family members in glioma

Since the IRF family may regulate glioma progression and prognosis by participating in a wide range of inflammatory and immune responses, we undertook a comprehensive analysis of tumor immune infiltrates using the TIMER database. The results are shown in Table 2. In LGG, the expression of all IRF family members was positively correlated with infiltration of B cells, CD4+ T cells, macrophages, neutrophils, and dendritic cells (all *p* < 0.01). Similar results were obtained for GBM. In addition, IRF1, IRF2, IRF6, and IRF9 were positively correlated with CD8+ T cell infiltration in LGG. However, IRF1, IRF5, IRF6, IRF7, IRF8, and IRF9 expression were negatively correlated with CD8+ T cell infiltration in GBM.

**Table 2:** Correlation between differentially expressed IRF family members and six types of tumor-infiltrating immune cells in LGG and GBM (TIMER).

**Gene**	**LGG**						**GBM**					
	**B_cell**	**CD8_T cell**	**CD4_T cell**	**Macrophage**	**Neutrophil**	**Dendritic**	**B_cell**	**CD8_T cell**	**CD4_T cell**	**Macrophage**	**Neutrophil**	**Dendritic**
IRF1	0.423	0.299	0.478	0.473	0.543	0.605	0.131	–0.201	0.024^†^	–0.022^†^	0.199	0.457
IRF2	0.483	0.323	0.514	0.62	0.51	0.506	0.083^†^	0.025^†^	0.15	0.180	0.26	0.281
IRF3	0.325	–0.072^†^	0.481	0.408	0.437	0.422	0.062^†^	0.049^†^	0.058^†^	0.149	0.125	0.175
IRF4	0.158	0.063^†^	0.311	0.242	0.282	0.274	0.050^†^	–0.119^†^	–0.151	–0.077	–0.167	–0.095^†^
IRF5	0.64	0.052^†^	0.886	0.784	0.761	0.819	0.116	–0.309	0.13	0.091^†^	0.098	0.134
IRF6	0.181	0.236	0.146	0.162	0.248	0.215	0.089^†^	–0.26	0.060^†^	0.140^†^	0.068 ^†^	0.103^a^
IRF7	0.333	0.010^†^	0.563	0.492	0.475	0.539	0.252	–0.167	0.096^†^	0.039^†^	0.143	0.214
IRF8	0.7	0.079^†^	0.792	0.631	0.683	0.77	0.253	–0.369	0.251	0.151	0.457	0.298
IRF9	0.293	0.144	0.401	0.361	0.323	0.427	0.248	–0.15	0.19	0.111	0.268	0.159

Note: ^†^*P* ≥ 0.05

We also examined correlations between clinical outcome, immune cell abundance, and IRF expression using the Cox proportional hazard model. After correcting for confounding factors, we found that B cells (*p* = 0.002), CD8+ T cells (*p* = 0.042), IRF1 expression (*p* = 0.001), and IRF8 expression (*p* = 0.000) were significantly associated with the prognosis of LGG patients, while CD4+ T cells, dendritic cells (*p* =0.002), IRF1 expression (*p* = 0.01), IRF7 expression (*p* = 0.007), and IRF8 expression (*p* = 0.028) were associated with the prognosis of GBM patients (Table 3).

**Table 3:** Cox proportional hazard model of IRF family members and six types of tumor-infiltrating immune cells in LGG and GBM (TIMER).

**Variable**	**LGG**						**GBM**					
	**Coef**	**HR**	**95%CI_L**	**95%CI_U**	***P*-value**	**Sig**	**Coef**	**HR**	**95%CI_L**	**95%CI_U**	***P*-value**	**Sig**
B_cell	10.843	51189.065	55.429	47273219.787	0.002	^**^	0.242	1.274	0.186	8.739	0.806	
CD8_Tcell	7.374	1593.545	1.284	1977201.523	0.042	^*^	–0.127	0.881	0.236	3.283	0.850	
CD4_Tcell	5.869	353.828	0.089	1399115.433	0.165		2.947	19.048	1.814	200.025	0.014	^*^
Macrophage	3.359	28.764	0.46	1792.334	0.111		0.540	1.715	0.137	21.455	0.675	
Neutrophil	–6.858	0.001	0.000	1.749	0.070		–0.977	0.377	0.017	8.373	0.537	
Dendritic	–2.367	0.094	0.002	5.295	0.250		2.005	7.426	2.131	25.882	0.002	^**^
IRF1	0.508	1.662	1.241	2.226	0.001	^**^	–0.536	0.585	0.389	0.881	0.010	^*^
IRF2	0.161	1.174	0.698	1.976	0.546		0.323	1.381	0.812	2.348	0.23	
IRF3	0.160	1.173	0.752	1.829	0.481		–0.021	0.980	0.554	1.732	0.944	
IRF4	–0.332	0.717	0.464	1.110	0.136		–0.059	0.943	0.298	2.978	0.920	
IRF5	0.157	1.170	0.695	1.972	0.555		0.239	1.270	0.830	1.943	0.271	
IRF6	–0.150	0.860	0.627	1.181	0.351		–0.301	0.740	0.491	1.115	0.150	
IRF7	0.344	1.411	0.980	2.030	0.064		0.545	1.725	1.159	2.566	0.007	^**^
IRF8	–0.755	0.470	0.326	0.678	0.000	^**^	–0.521	0.594	0.373	0.944	0.028	^*^
IRF9	–0.379	0.685	0.441	1.063	0.092		–0.201	0.818	0.503	1.331	0.419	

Note: ^*^P < 0.05, ^**^P <0.01

## DISCUSSION

IRF family members are important mediators of inflammatory and immune microenvironment signaling pathways that are necessary for cancer development and progression. Dysregulation of IRF family members has been observed in several types of malignancies, including leukemia [13], melanoma [14], breast cancer [15], and hepatocellular carcinoma [16]. However, there is a paucity of evidence regarding the overall role of IRFs in glioma. In this study, we set out to analyze the expression profile, prognostic value, and biological function of individual IRF members in glioma. The goal of the study was to advance our current understanding of the glioma microenvironment and to identify potential improvements in treatment strategies and prognostic accuracy for glioma patients.

We first explored the expression profiles of IRF family members and their correlations with pathological grade and patient outcomes. We found that IRF1, IRF2, IRF5, IRF7, IRF8, and IRF9 were upregulated in glioma compared with normal tissue. Moreover, IRF1, IRF2, IRF3, IRF4, IRF5, IRF7, IRF8, and IRF9 expression increased as tumors progressed, and glioma patients with low expression of these genes had significantly better overall survival. Some of these findings are consistent with previous results. For example, Liang et al. found that IRF1 expression was significantly elevated in glioma cell lines and IRF1 knockdown increased apoptosis and enhanced the efficacy of anti-VEGF therapy in an animal model of glioma [12]. In addition, Jin et al. reported that IRF7 was overexpressed in both glioma cell lines and human glioma specimens and was associated with reduced patient survival. Furthermore, IRF7 depletion could suppress glioma progression and decrease cellular heterogeneity *in vivo* through interleukin-6 and Notch signaling [10]. In contrast with our present results, a prior study by Dr. Tarassishin and colleagues reported that IRF3 inhibited glioma proliferation, migration, and invasion *in vitro* [11]. This inconsistency may be the result of inherent differences between transcriptomics studies and experimental validation studies or could reflect the heterogeneous nature of glioma, demonstrating the need for additional studies. Together, our study and previous studies suggest that differentially expressed IRF family members may play a role in glioma tumorigenesis and progression.

To explore the impact of IRF family member expression in glioma, we conducted a comprehensive analysis of patient characteristics and outcomes. We found that genetic alterations in IRFs were relatively uncommon in glioma, but patients with alterations exhibited more favorable overall survival, suggesting these changes may have a clinically significant impact on patient outcome. Furthermore, significant positive correlations were observed among the differentially expressed IRF family members, suggesting that these genes may play a synergistic role in the pathogenesis of glioma. The subsequent PPI network analysis also confirmed the close interactions among these genes.

We then examined the functions of differentially expressed IRF family members using multiple enrichment analysis tools. As expected, we found that these genes are mainly associated with the type I interferon signaling pathway, viral carcinogenesis, toll-like receptor signaling pathway, RIG-I-like receptor signaling pathway, and TGF-beta signaling pathway, all of which are primarily related to inflammation and immunity processes. Additionally, GSCALite analysis showed that IRF genes participate in a variety of biological processes including apoptosis, cell cycle, DNA damage response, and tumor-related pathways, such as the EMT, RAS/MAPK, RTK, and PI3K/AKT pathways. These findings strengthen the understanding of the biological mechanisms by which IRF family members participate in glioma pathology.

IRFs participate in the innate and adaptive immune responses of the body by regulating the development, migration, and localization of immune cells. Increasing evidence suggests that immune cell infiltration can influence the tumor microenvironment, thereby affecting tumor growth and progression and in turn playing significant roles in response to immunotherapy and patient outcome [17]. Microglia and macrophages, the main immune cell types that infiltrate gliomas, could facilitate glioma proliferation and migration by creating a supportive stroma and releasing several growth factors and cytokines [18]. Tumor-infiltrating lymphocytes, including CD4+ and CD8+ cells, are also found in glioma, and their levels are correlated with patient survival [19]. In this study, IRF expression was correlated with infiltration of the six immune cell types in glioma, including B cells, CD8+, CD4+ T cells, macrophages, and dendritic cells, and infiltration of some of these cells was independently associated with patient outcome. Our findings emphasized the important influence of IRF family member expression on immune cell infiltration in glioma.

Several limitations in our study should be considered when interpreting the results. First, because this is a retrospective study based on limited data from bioinformatic databases, we were unable to examine the specific roles of IRFs in different pathological types of glioma, such as astrocytoma or oligodendroglioma; future prospective studies with appropriate sample sizes are therefore warranted to expand our findings. Second, mRNA levels do not completely reflect protein levels in tumor tissues because of complex post-transcriptional regulation within cells. Further basic research is therefore necessary to further characterize the expression of and molecular mechanisms associated with IRF family members in glioma.

In summary, this study showed that IRF1, IRF2, IRF5, IRF8, and IRF9 mRNA levels were increased in glioma compared to normal tissue. Increased expression of IRF1, IRF2, IRF3, IRF4, IRF5, IRF7, IRF8, and IRF9 was associated with more advanced pathological grade and worse outcomes in glioma patients. Moreover, although genetic alterations in IRFs were relatively rare in glioma patients, they were associated with more favorable outcomes. Finally, IRF expression was correlated with immune cell infiltration in glioma. The results of the bioinformatics analyses performed in this study should be confirmed and expanded upon in future studies.

## MATERIALS AND METHODS

### Gene expression profile data and analysis

We used a two-step analysis to assess IRF family member expression patterns in glioma patients. First, we examined mRNA level data from ONCOMINE, the largest public microarray database for genome-wide expression analysis [20]. Data were extracted and compared to evaluate IRF family member expression in glioma specimens and normal controls under the "Brain and CNS Cancer" category. A *p*-value of 0.05, fold change of 2, and gene rank in the top 10% were selected as inclusion thresholds for the comparison. IRF family member expression data for different glioma subtypes, i.e., LGG and GBM, was then obtained from Gene Expression Profiling Interactive Analysis (GEPIA), another online tool that contains RNA sequence expression data from 518 LGG samples, 163 GBM samples, and 207 normal brain samples [21]. IRF expression was compared between LGG or GBM and normal tissues using Student *t*-tests; *p* < 0.05 and fold change >2 were considered significant.

### Clinicopathological correlation and prognosis analysis

Correlations between IRF family member expression and clinicopathological characteristics and prognosis in glioma patients were evaluated using data derived from The Cancer Genome Atlas (TCGA) database, which contains both sequencing and clinical records for over 30 types of human cancers [22]. A total of 527 patients with LGG (“LGG” dataset) and 167 patients with GBM (“GBM” dataset) were included in the analysis. IRF expression was compared in different tumor grades using one-way analysis of variance (ANOVA) following by Dunnett tests. Survival analysis was performed using Kaplan-Meier curves with samples divided into high- and low-expression groups according to median mRNA levels of each IRF. A log-rank *p*-value <0.05 was considered significant.

### Molecular characteristics and interaction analysis

The molecular characteristics and internal/external interactions of IRF family members were explored with multiple tools. Genetic alterations and their associations with patient prognosis were evaluated using cBioPortal, an online tool for visualization and analysis of multidimensional cancer genomics data [23]. Co-expression among IRF family members was evaluated in Pearson’s correlation tests using data from TCGA “LGG” and “GBM” datasets. The internal protein-protein interaction network among IRF family members was constructed and visualized using the STRING database [24]. The external interaction network between IRF family members and functionally related genes was generated using GeneMANIA [25].

### Functional enrichment analysis

To examine biological functions, Gene Ontology (GO) and Kyoto Encyclopedia of Genes and Genomes (KEGG) enrichment analyses were conducted for IRF family members and functionally related genes using DAVID software (version 6.8) [26]. Biological processes (BP), cellular components (CC), and molecular function (MF) categories were included in the GO enrichment analysis. The significance threshold was *p* < 0.05. Additionally, associations between IRF family members and the activity of cancer pathways, including TSC/mTOR, RTK, RAS/MAPK, PI3K/AKT, hormone ER, hormone AR, EMT, DNA damage response, cell cycle, and apoptosis pathways, were explored with GSCALite, a web-based platform for gene set cancer analysis [27].

### Tumor immune infiltrate analysis

The TIMER (Tumor Immune Estimation Resource) database is an immune infiltrate analysis tool for systematic evaluation of the different immune cells that infiltrate tumor tissue and their clinical significance [28]. In our study, we used the “Gene Module” to calculate correlations between the expression of each IRF and the abundance of infiltrating immune cells in glioma. Moreover, associations between patient outcomes and the abundance of immune infiltrates or gene expression were determined using “Survival module”.

## References

[1] Weller M, Wick W, Aldape K, Brada M, Berger M, Pfister SM, Nishikawa R, Rosenthal M, Wen PY, Stupp R, Reifenberger G. Glioma. Nat Rev Dis Primers. 2015; 1:15017. 10.1038/nrdp.2015.17 27188790

[2] Louis DN, Perry A, Reifenberger G, von Deimling A, Figarella-Branger D, Cavenee WK, Ohgaki H, Wiestler OD, Kleihues P, Ellison DW. The 2016 World Health Organization Classification of Tumors of the Central Nervous System: a summary. Acta Neuropathol. 2016; 131:803–20. 10.1007/s00401-016-1545-1 27157931

[3] Woehrer A, Bauchet L, Barnholtz-Sloan JS. Glioblastoma survival: has it improved? Evidence from population-based studies. Curr Opin Neurol. 2014; 27:666–74. 10.1097/WCO.0000000000000144 25364955

[4] Zhang X, Zhu S, Li T, Liu YJ, Chen W, Chen J. Targeting immune checkpoints in malignant glioma. Oncotarget. 2017; 8:7157–74. 10.18632/oncotarget.12702 27756892 PMC5351697

[5] Jacquelot N, Yamazaki T, Roberti MP, Duong CPM, Andrews MC, Verlingue L, Ferrere G, Becharef S, Vétizou M, Daillère R, Messaoudene M, Enot DP, Stoll G, et al. Sustained Type I interferon signaling as a mechanism of resistance to PD-1 blockade. Cell Res. 2019; 29:846–61. 10.1038/s41422-019-0224-x 31481761 PMC6796942

[6] Benci JL, Xu B, Qiu Y, Wu TJ, Dada H, Twyman-Saint Victor C, Cucolo L, Lee DSM, Pauken KE, Huang AC, Gangadhar TC, Amaravadi RK, Schuchter LM, et al. Tumor Interferon Signaling Regulates a Multigenic Resistance Program to Immune Checkpoint Blockade. Cell. 2016; 167:1540–54.e12. 10.1016/j.cell.2016.11.022 27912061 PMC5385895

[7] Honda K, Takaoka A, Taniguchi T. Type I interferon [corrected] gene induction by the interferon regulatory factor family of transcription factors. Immunity. 2006; 25:349–60. 10.1016/j.immuni.2006.08.009 16979567

[8] Tamura T, Yanai H, Savitsky D, Taniguchi T. The IRF family transcription factors in immunity and oncogenesis. Annu Rev Immunol. 2008; 26:535–84. 10.1146/annurev.immunol.26.021607.090400 18303999

[9] Chen YJ, Li J, Lu N, Shen XZ. Interferon regulatory factors: A key to tumour immunity. Int Immunopharmacol. 2017; 49:1–5. 10.1016/j.intimp.2017.05.010 28531759

[10] Jin X, Kim SH, Jeon HM, Beck S, Sohn YW, Yin J, Kim JK, Lim YC, Lee JH, Kim SH, Kang SH, Pian X, Song MS, et al. Interferon regulatory factor 7 regulates glioma stem cells via interleukin-6 and Notch signalling. Brain. 2012; 135:1055–69. 10.1093/brain/aws028 22434214

[11] Tarassishin L, Lee SC. Interferon regulatory factor 3 alters glioma inflammatory and invasive properties. J Neurooncol. 2013; 113:185–94. 10.1007/s11060-013-1109-3 23512614

[12] Liang J, Piao Y, Henry V, Tiao N, de Groot JF. Interferon-regulatory factor-1 (IRF1) regulates bevacizumab induced autophagy. Oncotarget. 2015; 6:31479–92. 10.18632/oncotarget.5491 26362401 PMC4741619

[13] Deng M, Daley GQ. Expression of interferon consensus sequence binding protein induces potent immunity against BCR/ABL-induced leukemia. Blood. 2001; 97:3491–97. 10.1182/blood.v97.11.3491 11369642

[14] Fratta E, Sigalotti L, Covre A, Parisi G, Coral S, Maio M. Epigenetics of melanoma: implications for immune-based therapies. Immunotherapy. 2013; 5:1103–16. 10.2217/imt.13.108 24088079

[15] Connett JM, Badri L, Giordano TJ, Connett WC, Doherty GM. Interferon regulatory factor 1 (IRF-1) and IRF-2 expression in breast cancer tissue microarrays. J Interferon Cytokine Res. 2005; 25:587–94. 10.1089/jir.2005.25.587 16241857

[16] Gao Q, Zhu H, Dong L, Shi W, Chen R, Song Z, Huang C, Li J, Dong X, Zhou Y, Liu Q, Ma L, Wang X, et al. Integrated Proteogenomic Characterization of HBV-Related Hepatocellular Carcinoma. Cell. 2019; 179:561–77.e22. 10.1016/j.cell.2019.08.052 31585088

[17] Sampson JH, Gunn MD, Fecci PE, Ashley DM. Brain immunology and immunotherapy in brain tumours. Nat Rev Cancer. 2020; 20:12–25. 10.1038/s41568-019-0224-7 31806885 PMC7327710

[18] Hambardzumyan D, Gutmann DH, Kettenmann H. The role of microglia and macrophages in glioma maintenance and progression. Nat Neurosci. 2016; 19:20–27. 10.1038/nn.4185 26713745 PMC4876023

[19] Liu Z, Meng Q, Bartek J Jr, Poiret T, Persson O, Rane L, Rangelova E, Illies C, Peredo IH, Luo X, Rao MV, Robertson RA, Dodoo E, Maeurer M. Tumor-infiltrating lymphocytes (TILs) from patients with glioma. Oncoimmunology. 2016; 6:e1252894. 10.1080/2162402X.2016.1252894 28344863 PMC5353900

[20] Rhodes DR, Kalyana-Sundaram S, Mahavisno V, Varambally R, Yu J, Briggs BB, Barrette TR, Anstet MJ, Kincead-Beal C, Kulkarni P, Varambally S, Ghosh D, Chinnaiyan AM. Oncomine 3.0: genes, pathways, and networks in a collection of 18,000 cancer gene expression profiles. Neoplasia. 2007; 9:166–80. 10.1593/neo.07112 17356713 PMC1813932

[21] Tang Z, Li C, Kang B, Gao G, Li C, Zhang Z. GEPIA: a web server for cancer and normal gene expression profiling and interactive analyses. Nucleic Acids Res. 2017; 45:W98–102. 10.1093/nar/gkx247 28407145 PMC5570223

[22] Weinstein JN, Collisson EA, Mills GB, Shaw KR, Ozenberger BA, Ellrott K, Shmulevich I, Sander C, Stuart JM, and Cancer Genome Atlas Research Network. The Cancer Genome Atlas Pan-Cancer analysis project. Nat Genet. 2013; 45:1113–20. 10.1038/ng.2764 24071849 PMC3919969

[23] Gao J, Aksoy BA, Dogrusoz U, Dresdner G, Gross B, Sumer SO, Sun Y, Jacobsen A, Sinha R, Larsson E, Cerami E, Sander C, Schultz N. Integrative analysis of complex cancer genomics and clinical profiles using the cBioPortal. Sci Signal. 2013; 6:pl1. 10.1126/scisignal.2004088 23550210 PMC4160307

[24] Szklarczyk D, Gable AL, Lyon D, Junge A, Wyder S, Huerta-Cepas J, Simonovic M, Doncheva NT, Morris JH, Bork P, Jensen LJ, Mering CV. STRING v11: protein-protein association networks with increased coverage, supporting functional discovery in genome-wide experimental datasets. Nucleic Acids Res. 2019; 47:D607–13. 10.1093/nar/gky1131 30476243 PMC6323986

[25] Warde-Farley D, Donaldson SL, Comes O, Zuberi K, Badrawi R, Chao P, Franz M, Grouios C, Kazi F, Lopes CT, Maitland A, Mostafavi S, Montojo J, et al. The GeneMANIA prediction server: biological network integration for gene prioritization and predicting gene function. Nucleic Acids Res. 2010; 38:W214–20. 10.1093/nar/gkq537 20576703 PMC2896186

[26] Huang W, Sherman BT, Lempicki RA. Systematic and integrative analysis of large gene lists using DAVID bioinformatics resources. Nat Protoc. 2009; 4:44–57. 10.1038/nprot.2008.211 19131956

[27] Liu CJ, Hu FF, Xia MX, Han L, Zhang Q, Guo AY. GSCALite: a web server for gene set cancer analysis. Bioinformatics. 2018; 34:3771–72. 10.1093/bioinformatics/bty411 29790900

[28] Li T, Fan J, Wang B, Traugh N, Chen Q, Liu JS, Li B, Liu XS. TIMER: A Web Server for Comprehensive Analysis of Tumor-Infiltrating Immune Cells. Cancer Res. 2017; 77:e108–10. 10.1158/0008-5472.CAN-17-0307 29092952 PMC6042652

